# Supplementary material for: A qualitative case study of community experiences with Tungiasis in high prevalence villages of Bungoma County, Kenya: “The whole body aches and the jiggers are torturing me!”
Source: PLoS Negl Trop Dis. 2023 Apr 26;17(4):e0011304. doi: 10.1371/journal.pntd.0011304 (PMC10166527; doi:10.1371/journal.pntd.0011304)
Supplement: Attachment — Submitted filename: renamed_00bb5.docx [file pntd.0011304.s001.docx]

PLOS Neglected Tropical Diseases submission (PNTD-D-22-01214)

## Title of manuscript: “*The whole body aches and the jiggers are torturing me!”:* A qualitative study of community experiences with Tungiasis, in a high prevalence rural area Bungoma, Western Kenya

### Authors: Mørkve, Åse Walle, Van den Bergh, Graziella, Sitienei, Judith

Thank you for the invitation to resubmission of a significantly-revised version for this article.

I have made the following changes:

- Revised the article according to SRQR checklist
- Separated the Results and Discussion section
- Added citations, pictures and changed themes in the findings section, in order to better highlight the main findings.
- Added a table with participants’ information, as required.
