# Supplementary material for: A qualitative case study of community experiences with Tungiasis in high prevalence villages of Bungoma County, Kenya: “The whole body aches and the jiggers are torturing me!”
Source: PLoS Negl Trop Dis. 2023 Apr 26;17(4):e0011304. doi: 10.1371/journal.pntd.0011304 (PMC10166527; doi:10.1371/journal.pntd.0011304)
Supplement: Attachment — Submitted filename: PLOS 22.03 response to reviewers .docx [file pntd.0011304.s002.docx]

**Response to reviewers**

**As a result of the suggested changes by editor/ reviews, the abstract and method chapter is significantly revised.**

| **Suggested changes by editor/ reviewers** | **Comment from Authors** |
| --- | --- |
| Consider the modification of the title emphasizing a clear objective of the study, with study design and location. I would suggest to avoid initial statement in the title of this manuscript. | Line 1-2 |
| Provide clear information on how the interview guide was prepared | Line 153 - 159 |
| Provide the details of number of samples included in this study, along with total number of themes extracted from qualitative analysis at the start of results. | Line 195 - 197 |
| The first sentence of the limitation section is not clear, rather than to mention first and second author, the authors should just describe the major limitations, if one limitation is addressed by any of the authors, it would not be considered as limitation. | Line 461 - 471 |
| I recommend that authors consider having their work edited by a professional service or a native English speaker. | Done |
| Methods: Although the study design has been well described, the aim of the survey could be formulated a little more clearly (especially in the abstract). It is also not entirely clear according to which criteria the study participants were selected. | Line 31 – 33  Line 160 - 173 |
| Results: Does the analysis presented match the analysis plan? - Partly | Line 174 - 185  Figure 3 and 4 |
| Results: Are the figures (Tables, Images) of sufficient quality for clarity? - NO | Revision of Figure 2,3 and 4 |
| Conclusion: Perhaps the recommendations for action in practice could be formulated more clearly (as a list?) in order to give the topic more weight. | Line 485 - 517 |
| There is the absence of an appropriate legend in table 1, in the topics defined by the asterisks, which are inserted in the table itself. As a suggestion, the information should appear as a legend and not as an integral part in one more line of the table itself. | Figure 2 |
| The variables used for data collection can be described more clearly, with the construction of a table or a flowchart, demonstrating the structure of the topics used, including the "key words" captured by the recording system. | Figure 4 |
| As a suggestion, in addition to clinical illustrative images, due to the severe environmental context described in the results, if available, it would be essential to insert images of the households and the surroundings, emphasizing the sanitary problems that permeate the occurrence and recurrence of tungiasis in this population studied. | Figure 7 |
| It is very clear that the authors draw attention to the relevance of the social context and poverty that involve the population affected by tungiasis; however, while a world with more equity and social justice does not emerge as the solution to almost all the problems that afflict humanity, it is necessary to think of strategies that can collaborate to the improvement of the health conditions of these people. | Line 485 - 517 |
| In this aspect, this reviewer missed the authors' mention of therapeutic strategies that may be promising, such as the use of occlusive agents, especially manufactured dimethicone-based products, as mentioned in a systematic review. In another study, Schwalfenberg et al. (2004) demonstrated in a small case series that the use of coconut and jojoba oil in natura may be useful in avoiding reinfection [1, 2]. | Line 428 - 435 |
